# Recurrent postoperative delirium in spinocerebellar ataxia type 2: a case report

**DOI:** 10.1186/s13256-019-2040-9

**Published:** 2019-04-29

**Authors:** Laura Levantesi, Germano De Cosmo, Giandomenico Logroscino, Michela Saracco

**Affiliations:** 1 Department of Anaesthesiology and Intensive Care Medicine, Università Cattolica del Sacro Cuore - Fondazione Policlinico Universitario A. Gemelli IRCCS, Rome, Italy; 2 Department of Anaesthesiology and Intensive Care Medicine, Fondazione Policlinico Universitario A. Gemelli IRCCS, Largo Agostino Gemelli 1, 00168 Rome, Italy; 3 Mininvasive and Computer Assisted Orthopaedic Surgery, University of L’Aquila, L’Aquila, Italy; 4 Department of Orthopaedics, Università Cattolica del Sacro Cuore - Fondazione Policlinico Universitario A. Gemelli IRCCS, Rome, Italy

**Keywords:** General anesthesia, Delirium, Mental disorder, Spinocerebellar ataxia, Hip arthroplasty

## Abstract

**Background:**

Postoperative delirium is a relatively uncommon condition in middle aged patients, but very widespread in patients with psychiatric and neurological diseases undergoing general anesthesia. Few studies are currently available in the literature on the perioperative anesthesiological management of patients suffering from spinocerebellar ataxia.

**Case presentation:**

A 58-year-old Caucasian woman affected by spinocerebellar ataxia type 2 underwent total hip arthroplasty for advanced osteoarthritis. One month later, debridement, antibiotics, and implant retention was performed for periprosthetic hip infection. Both times she underwent general anesthesia and developed an early postoperative delirium treated successfully with chlorpromazine.

**Conclusions:**

This case report highlights the need to correctly manage patients at high risk of developing postoperative delirium, especially if suffering from degenerative neurological diseases. On the other hand, further studies will be needed in order to evaluate if spinocerebellar ataxia is an independent risk factor for the development of this acute and transient pathological condition.

## Introduction

Ataxia is characterized by a lack of voluntary coordination of muscle movements. There are different types of ataxia according to onset and progress. In addition, the clinical features of most ataxias overlap. Therefore, there is a need to typify the genotype in order to predict the course of the specific disease. Spinocerebellar ataxias (SCAs) are a heterogeneous group of autosomal dominant cerebellar ataxias. SCAs have been classified into 43 subtypes based on the genetic mutations that characterize them. Spinocerebellar ataxia type 2 (SCA2) is the second most common disorder and one of the most severe subtypes, affecting middle aged patients. It is characterized by slowly progressive ataxia and dysarthria associated with nystagmus, slow saccadic eye movements and, in some individuals, ophthalmoparesis or parkinsonism. On the other hand, hypotonia is uncommon. Mutations in the *ATXN2* gene cause SCA2. This gene provides instructions for making a protein called ataxin-2. The *ATXN2* gene mutation that causes SCA2 involves a DNA segment known as a CAG trinucleotide repeat. Individuals with an abnormally long CAG segment develop this neurological disease [1]. Thus, diagnosis depends on molecular genetic testing. In addition, morphometric magnetic resonance imaging (MRI) shows a severe olivopontocerebellar atrophy pattern and frontal cortex atrophy [2]. Patients with SCA2 are often affected by peripheral neuronopathy with hypo/areflexia, paresthesia, and hypoesthesia. Cognitive performances are characterized by frontal-executive dysfunctions, verbal memory impairments, attentional deficits and, sometimes, dementia. Psychiatric symptoms include depression in 22% of cases and anxiety. People with SCA2 usually survive 10 to 20 years after symptoms appear [3].

In this case report a patient suffering from SCA2 underwent two different orthopedic surgical procedures under general anesthesia and developed early postoperative delirium (POD). Several reports have been published on the anesthetic management of patients with SCA, but few of these deal with the management of patients affected by the subtype SCA2 and none addresses prevention and management of the POD.

## Case presentation

A 58-year-old Caucasian woman with hip osteoarthritis was examined by an anesthesiologist for a surgical procedure of total hip arthroplasty. She had a history of SCA, which started when she was 55-years old with motor dysfunction. Her clinical condition had slowly worsened with appearance of dysarthria, horizontal saccadic eye movements, and lower extremities hypertonia. An MRI of her brain showed olivopontocerebellar atrophy; a mild motor and sensory ataxic polyneuropathy was highlighted by electromyography. Recent neuropsychological examinations identified a dis-executive deficit. Her family history is negative for SCA and she denied any history of cardiovascular, respiratory, or gastrointestinal diseases. Prior to the diagnosis of SCA, she was in good health and did not regularly take drugs. She denied smoking tobacco or drinking alcohol. At the time of the examination, she was taking benzodiazepines (triazolam 0.25 mg once daily) for anxious depressive syndrome, baclofen 25 mg three times a day for spasticity, and anti-cyclooxygenase type 2 (COX-2) for pain treatment (etoricoxib 60 mg once daily). Preoperative blood tests, electrocardiogram, and thoracic X-ray were negative. On physical examination, it was possible to appreciate that she was tall, 168 cm, and weighed 63 kg. A cardiopulmonary examination was unremarkable. Her vital signs were normal with blood pressure of 135/90 mmHg and heart rate of 90 beats per minute. On neuropsychological examination, she presented a mild reduction in performance on the Rey–Osterrieth Complex Figure Test, a limited ability to inhibit cognitive interference (Stroop Test), inability during the Multiple Features Targets Cancellation task, and a Spatial Span Score lower than normal; these were proofs of her dis-executive deficit. After discussing the case with a neurologist, general anesthesia was planned. In fact, our patient’s anxiety and spasticity would have made regional anesthesia difficult to practice. Preoperatively, no medications were administered. General anesthesia was induced with propofol 2 mg/kg intravenously and fentanyl 2 mcg/kg intravenously. Tracheal intubation was facilitated with rocuronium 0.6 mg/kg intravenously. Anesthesia maintenance was performed with sevoflurane (in an oxygen/air mixture) and fentanyl. The minimum alveolar concentration of sevoflurane was set to achieve a Bi-spectral Index (BIS) value between 40 and 60. Mechanical ventilation was set with a tidal volume of 7 ml/kg, a positive end-expiratory pressure (PEEP) of 5 cmH_2_O, and an inspiration/expiration rate of 1:2. These parameters remained stable during the surgical procedure. The respiration rate was adjusted to obtain an end-tidal carbon dioxide (etCO_2_) between 38 and 45 mmHg. The surgery was uneventful with no significant blood loss and no transfusion was needed. The procedure lasted 2 hours. Upon awakening, she appeared calm and parameters were stable. In the post anesthesia care unit (PACU), she was warmed up and oxygen supplementation was administered. Thirty minutes after awakening, she developed an acute hyperactive delirium. She experienced episodes of confusion, logorrhea, and disorientation. She also exhibited reduced awareness of the environment and hallucinations with aggressive behavior. During this episode, her vital signs remained stable and her oxygen saturation was normal. An arterial blood test revealed no hypercapnia or hypoxia or electrolyte disorders. Haloperidol 5 mg was intravenously administered slowly over 5 minutes, but her behavior did not change. To avoid uncontrolled pain, a single shot fascia iliaca block was performed with 25 ml of ropivacaine 0.375% but she continued to have intermittent episodes of delirium. After 10 minutes, chlorpromazine 25 mg was infused intravenously. This drug seemed to have the same efficacy as haloperidol with a subsequent mild sedation. She was transferred to our intensive care unit (ICU) where chlorpromazine 25 mg was given again during the night for another crisis. She was discharged from ICU the next day under normal neurological conditions.

After 1 month, she was readmitted for the development of an acute periprosthetic hip infection sustained by *Staphylococcus epidermidis* and *Staphylococcus haemolyticus* without signs of systemic sepsis. On examination, her temperature was 36.7°C, blood pressure was 128/95 mmHg, and heart rate was 75 beats per minute. Only the wound swab was positive for infection; hemocultures and urine cultures were negative. Preoperative blood tests showed: hemoglobin 10.4 g/dL; platelets 233 × 10^9^/L; neutrophils 4.26 × 10 ^9^/L; erythrocyte sedimentation rate (ESR) 47 mm/hour, and C-reactive protein (CRP) 27.2 mg/L. Renal and hepatic function indices were within normal limits. Debridement, antibiotics, and implant retention (DAIR) was performed under general anesthesia with the same modalities [4]. During surgery and after taking new periprosthetic tissue for microbiological examination, teicoplanin was administered intravenously at a loading dose of 400 mg. For the second time, our patient developed POD treated with chlorpromazine 25 mg intravenously with immediate sedation. Within 24 hours, the delirium resolved with no consequences. During the following days, 200 mg of teicoplanin was administered daily and 1 gr of cefazolin every 12 hours intravenously until discharge.

One month after discharge, she returned for follow-up. She was taking her habitual medications and, at the time of the examination, she exhibited no evidence of new disorders or joint infection. She looked well and denied any symptoms of confusion, disorientation, or hallucinations. The hip infection healed without any complication. She was re-evaluated at 3 and 6 months after surgery. Her ESR, CRP, and other biochemical values were within normal limits and her neurological status was stable.

## Discussion

There is a large literature on the anesthetic management of patients with SCA but this case report focuses on SCA2 and reports an anesthesia complication, POD, which is uncommon in middle aged people. An age of over 65 years, conventionally, represents a factor that can predispose or precipitate delirium. In general, patients with SCA2 may develop significant complications during and after general anesthesia, such as seizures, cardiomyopathy, and heart blocks. Non-cerebellar manifestations in SCA2 are oculomotor disturbances, signs of corticospinal tract dysfunctions (hyperreflexia and spasticity), and signs of neuronal degeneration, but also peripheral neuronopathy with hypo/areflexia, paresthesia, and hypoesthesia. Some forms of SCA2 present also a parkinsonian phenotype with dystonia, myoclonus, rigidity, and chorea. Common symptoms are also psychiatric disturbances, sleep disorders, and cognitive decline but dementia is rare. Although with less frequency, autonomic dysfunctions may appear, including urogenital, cardiovascular, gastrointestinal, and thermoregulatory disorders. Cardiovascular autonomic dysfunctions with involvement of sympathetic and parasympathetic system may occur in 60% of patients with SCA2 [3]. Patients with spinocerebellar degeneration have been successfully managed under regional and general anesthesia but tremors or rigidity may make performing the procedure difficult. However, autonomic neuropathy makes unpredictable responses after central neuraxial blockade. Moreover, neuropathy may worsen after performing regional anesthesia. On the other hand, general anesthesia exposes the patient to the risk of unpredictable responses to curare derivatives with prolonged paralysis and necessity of mechanical ventilation. However, Schmitt *et al.* reported the use of rocuronium in two children with Friedreich’s ataxia with no increase in onset and recovery timings [5]. In addition, various atrioventricular blocks have been reported after general anesthesia [6]. Our patient’s clinical conditions made general anesthesia preferable; in fact, no complications occurred during induction and maintenance of general anesthesia, but an early POD occurred. The incidence of POD in non-cardiac surgery is actually between 15 and 54% [7]. This condition seems to be caused by the release of psychoactive inflammatory markers during surgery, such as interleukin-6 (IL-6), tumor necrosis factor-alpha (TNF-alpha), and interleukin-1 (IL-1) beta, that contribute to delirium through dopamine, gamma-aminobutyric acid (GABA), or cholinergic-mediated pathways. General anesthesia, in addition, exposes the brain to cognitively active drugs.

*Diagnostic and Statistical Manual of Mental Disorders*, Fifth Edition (DSM-5) criteria for delirium are:

1. A disturbance in attention and awareness.
2. Development over a short period.
3. An additional disturbance in cognition (memory deficit, disorientation).
4. The disturbances in Criteria A and C are not explained by pre-existing neurocognitive disorder.
5. There is no evidence that the disturbance is a direct physiological consequence of another medical condition, substance intoxication or withdrawal (due to a drug of abuse or to a medication), or exposure to a toxin, or is due to multiple etiologies.

Therefore, it is important to implement optimal management and prevention strategies. First, the patient’s vital signs should be checked and an arterial blood test should be performed with a complete physical examination in order to exclude hypoxia and hypercarbia, hypoglycemia, and electrolyte disturbances but also stroke, seizures, and central cholinergic syndrome. Following the results of a recent meta-analysis, delirium treatment is multicomponent and antipsychotics are useful in the treatment and prevention of this complication. So, the treatment of delirium is based mainly on the use of typical antipsychotics like haloperidol [8]; however, recent findings suggest that second-generation antipsychotics are suitable for treating delirium and have a better safety profile than haloperidol [9]. In addition, adequate pain control plays a central role in this setting, using epidural analgesia, long-acting morphine and fascia iliaca block. Hydration and early mobilization are also important in preventing recurrence [10].

## Conclusions

According to the literature, POD is associated with significantly worse outcomes with an increase in adverse events, hospitalization, and mortality [11]. Multidisciplinary prevention programs have been shown to reduce the incidence of delirium among medical and surgical patients by approximately 30% [12]. In conclusion, the best treatment for POD is prevention, which needs recognition and management of risk factors but also close collaboration between surgeons, anesthesiologists, geriatricians, and neurologists in order to reduce the hospitalization. In conclusion, if identifying risk factors is mandatory to prevent this disease, then further studies are needed to evaluate if SCA is an independent risk factor for POD. In fact, it is possible that our patient’s anxious syndrome played a relevant role in the development of this acute neurological disorder.

## Abbreviations

BIS: Bi-spectral Index
COX-2: Cyclooxygenase type 2
CRP: C-reactive protein
DAIR: Debridement, antibiotics, and implant retention
DSM-5: *Diagnostic and Statistical Manual of Mental Disorders*, Fifth Edition
ESR: Erythrocyte sedimentation rate
etCO_2_: End-tidal carbon dioxide
GABA: Gamma-aminobutyric acid
ICU: Intensive care unit
Il-1: Interleukin-1
IL-6: Interleukin-6
MRI: Magnetic resonance imaging
PACU: Post anesthesia care unit
PEEP: Positive end-expiratory pressure
POD: Postoperative delirium
SCA: Spinocerebellar ataxia
SCA2: Spinocerebellar ataxia type 2
TNF-alpha: Tumor necrosis factor-alpha
